# Realizing Broadband and Invertible Linear-to-circular Polarization Converter with Ultrathin Single-layer Metasurface

**DOI:** 10.1038/srep18106

**Published:** 2015-12-15

**Authors:** Zhancheng Li, Wenwei Liu, Hua Cheng, Shuqi Chen, Jianguo Tian

**Affiliations:** 1 Laboratory of Weak Light Nonlinear Photonics, Ministry of Education, School of Physics and Teda Applied Physics Institute, Nankai University, Tianjin 300071, China

## Abstract

The arbitrary control of the polarization states of light has attracted the interest of the scientific community because of the wide range of modern optical applications that such control can afford. However, conventional polarization control setups are bulky and very often operate only within a narrow wavelength range, thereby resisting optical system miniaturization and integration. Here, we present the basic theory, simulated demonstration, and in-depth analysis of a high-performance broadband and invertible linear-to-circular (LTC) polarization converter composed of a single-layer gold nanorod array with a total thickness of ~*λ*/70 for the near-infrared regime. This setup can transform a circularly polarized wave into a linearly polarized one or a linearly polarized wave with a wavelength-dependent electric field polarization angle into a circularly polarized one in the transmission mode. The broadband and invertible LTC polarization conversion can be attributed to the tailoring of the light interference at the subwavelength scale via the induction of the anisotropic optical resonance mode. This ultrathin single-layer metasurface relaxes the high-precision requirements of the structure parameters in general metasurfaces while retaining the polarization conversion performance. Our findings open up intriguing possibilities towards the realization of novel integrated metasurface-based photonics devices for polarization manipulation, modulation, and phase retardation.

The polarization state of optical waves, which cannot be detected by human eyes, forms an important characteristic of such waves. The ability to manipulate the polarization state of optical waves can enable us to control light for a wide range of applications such as polarization manipulation, optical sensing, photography, and communication^1^,^2^,^3^. Conventional approaches to manipulate the polarization state of optical waves employ bulky waveplates, which are made of birefringent materials composed of crystalline solids and liquid crystals. However, the inherent disadvantages in the size, collimation, and bandwidth of these configurations prevent optical system miniaturization and integration.

Recently, plasmonic metasurfaces or metamaterials have been reported to provide a promising pathway towards the realizing of efficient polarization conversion via ultrathin, miniaturized, and easily integrable designs^4^,^5^,^6^,^7^,^8^,^9^,^10^,^11^. High-performance metamaterial-based linear polarization converters have been realized for both transmission and reflection modes in the gigahertz^12^,^13^,^14^, terahertz^15^,^16^,^17^, infrared^18^,^19^,^20^,^21^,^22^, and optical frequency regimes^23^,^24^. The functionality of broadband, high-efficiency half-wave plates has also been realized in patterned metal film and biperiodic gratings 25,26. Compared to linear polarization conversion, conversion between circularly polarized waves or between circularly and linearly polarized waves has attracted growing attention in the scientific community for potential application in biosensing and imaging. Broadband circular polarization conversion has been achieved with 3D optical metamaterials^27^,^28^. Further, the functionality of broadband and high-efficiency quarter-wave plates in the reflection mode has been realized in gap-plasmon resonators and plasmonic metasurfaces^29^,^30^. Thus far, from the perspective of practical application, considerable effort has been devoted to realize broadband and high-performance linear-to-circular (LTC) and circular-to-linear (CTL) polarization conversion in the transmission mode. The functionality of infrared broadband quarter-wave plates has been realized in the transmission mode in anisotropic Bézier metasurfaces^31^. Moreover, broadband optical meta-waveplates for CTL polarization conversion in the transmission mode have been proposed for the near-infrared regime via the use of plasmonic nanorods with two orthogonally coupled nanodipole elements^32^,^33^. A high-efficiency, broadband, tunable, and flexible quarter-wave plate based on a multilayer metamaterial also has been proposed for terahertz operation^34^. More recently, wideband LTC polarization conversion has been achieved with the use of an ultrathin bi-layered metasurface^35^. However, most of these designs for LTC or CTL polarization conversion in the transmission mode have complicated structural parameters, and their performance is strongly dependent on the resonance of each isolated building block or complex resonances between two layers, which leads to high-precision requirements of the structural parameters in order to ensure satisfactory polarization conversion performance. Therefore, these designs require highly advanced fabrication techniques, which requirement makes it difficult to implement such designs in integrated metasurface-based photonics systems.

Here, we present a new approach to realize the manipulation of the polarization state due to the tailoring of the light interference at the subwavelength scale by introducing the anisotropic optical resonance mode of the metasurface nanorod. We realize broadband and lossless LTC and CTL polarization converter simultaneously by using an ultrathin single-layer metasurface. Simulation results indicate that the ideal CTL polarization conversion can be realized in the wavelength range of 1100 nm to 2000 nm with a transmission intensity of more than 40%, and further, ideal LTC polarization converter can be achieved in the wavelength range of 1170 nm to 1590 nm with a transmission intensity of more than 30%. Further analysis reveals that the proposed ultrathin metasurface relaxes the high-precision requirements of the structure parameters in general metasurfaces, which may have a profoundly positive impact on its practical applications.

## Theory Analysis

We first consider the theory analysis of the LTC polarization converter. Let us consider an incoming plane wave that propagates along the +*z* direction, whose electric field can be expressed as


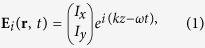


where 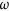 represents its frequency, 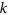 the wave vector, and 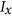 and 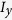 the complex amplitudes. Parameter 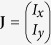, which is known as the Jones vector, determines the state of polarization and the total intensity of the wave. The Jones vectors of linearly polarized and right- and left-circularly polarized waves can be written as 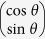, 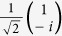, and 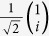, respectively, with 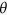 representing the angle between the *x*-axis and the electric component of the linearly polarized wave. The transmitted electric field can subsequently be described as


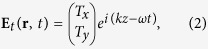


The incident and the transmitted electric fields are related via the Jones matrix **T**^36^,^37^, and this relation can be expressed as


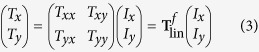


The indices *f* and lin indicate propagation in the forward (+*z* direction) and a special linear base with base vectors parallel to the coordinate axes, respectively. For a medium that has no linear polarization conversion effect (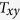 and 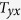 equal to zero), the transmitted field can be expressed as


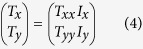


From equation (4) and the form of the Jones vectors of linearly polarized and right- and left-circularly polarized waves, it can be determined that for a high-performance LTC polarization converter with a linearly polarized wave 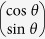, the phase difference between 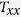 and 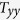 must be equal to 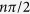, where *n* represents an odd number. Further, the condition 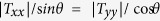 must be satisfied. Moreover, for the CTL polarization converter, only the phase difference between 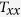 and 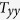 needs to be equal to 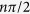, where *n* also denotes an odd number, which means that an LTC polarization converter can be used as a CTL converter.

### Design and characteristics

**Figure 1:**
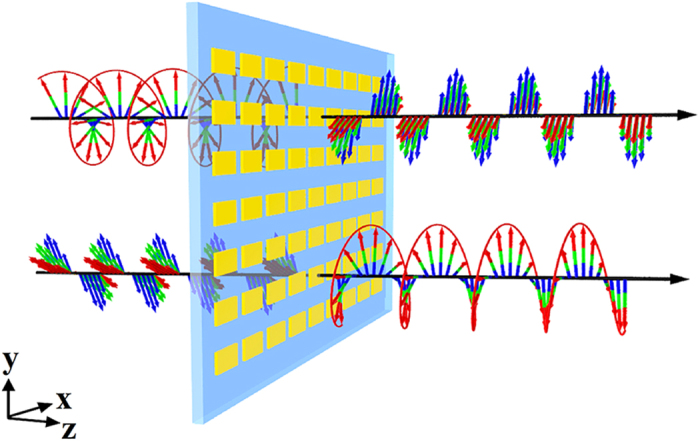
Artistic rendering of the proposed ultrathin metasurface. The device can transform a circularly polarized wave into a linearly polarized one or a linearly polarized wave with a wavelength-dependent electric field polarization angle into a circularly polarized one in the transmission mode.

To satisfy the abovementioned conditions and realize a high-performance broadband and invertible LTC polarization converter, we make use of the anisotropic optical resonance mode of a metasurface nanorod. Here, we propose the use of a single-layer gold nanorod array, whose thickness is 1/70 times the wavelength of interest, to realize the desired converter. Figure 1 shows the schematic of the designed ultrathin metasurface, wherein SiO_2_ is used as the substrate and the gold nanorod is designed to be 20 nm in thickness with a 20-nm-thick SiO_2_ layer covering the nanorod. The SiO_2_ covering on the nanorod prevents light interference at the interface of air and SiO_2_, which is induced by the anisotropic optical resonance mode of the single nanorod. It is beneficial to tune the anisotropic optical resonance mode to meet the phase condition. The periods of the unit-cell are all *P* = 620 nm along the *x* and *y* directions while the designed nanorod has dimensions of length *L* = 560 nm and width *W* = 290 nm.

In order to study this ultrathin metasurface, we conducted numerical simulations, which were carried out with the use of the CST Microwave Studio software package^38^. In our simulations, the refractive index of SiO_2_ was set to 1.47. The dielectric function of gold was defined by the Drude mode with plasmon frequency 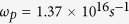 and damping constant 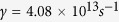 39. To account for surface scattering, grain boundary effects in the thin gold film, and inhomogeneous broadening, we used a damping constant that was three times greater than that of the bulk^40^,^41^,^42^. Periodic boundary conditions were set along the *x* and *y* directions representing a periodical structure, and an open (perfectly matched layer) boundary was defined in *z* direction for light-wave incidence and transmission.

**Figure 2:**
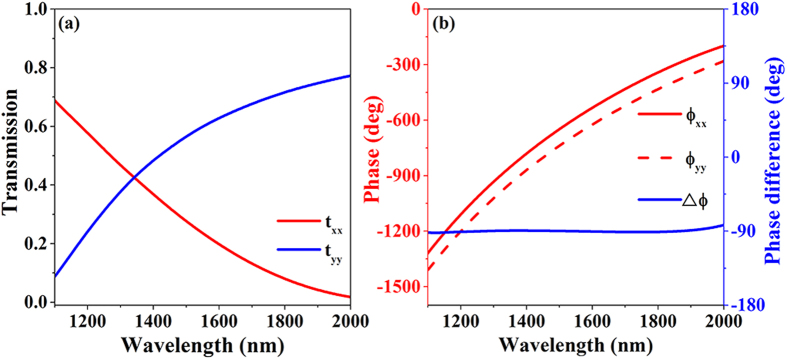
(**a**) Simulated squared moduli 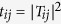 and (**b**) simulated phase difference of coefficients 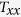 and 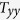 for linearly polarized excitations for the ultrathin metasurface geometry shown in Fig. 1.

Before analyzing the performance of the broadband and invertible LTC polarization converter, we determined the optical properties of the gold nanorod for a normally incident linearly polarized wave with its polarization direction along the *x*- and *y*-axes in that order. The simulated squared moduli 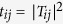 of the transmission coefficients 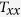 and 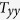 are shown in Fig. 2(a) as functions of the wavelength. The orthogonal optical resonance modes are excited at higher and lower wavelengths. The gold nanorod appears to act as a highly dispersive anisotropic optical resonator. Figure 2(b) depicts the phase difference 
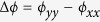
 between the transmission coefficients 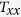 and 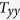, and this phase difference is exactly equal to 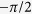 in the wavelength range from 1100 to 2000 nm, thereby fulfilling the phase condition required for device operation as an ideal ultrathin high-performance broadband CTL polarization converter.

**Figure 3:**
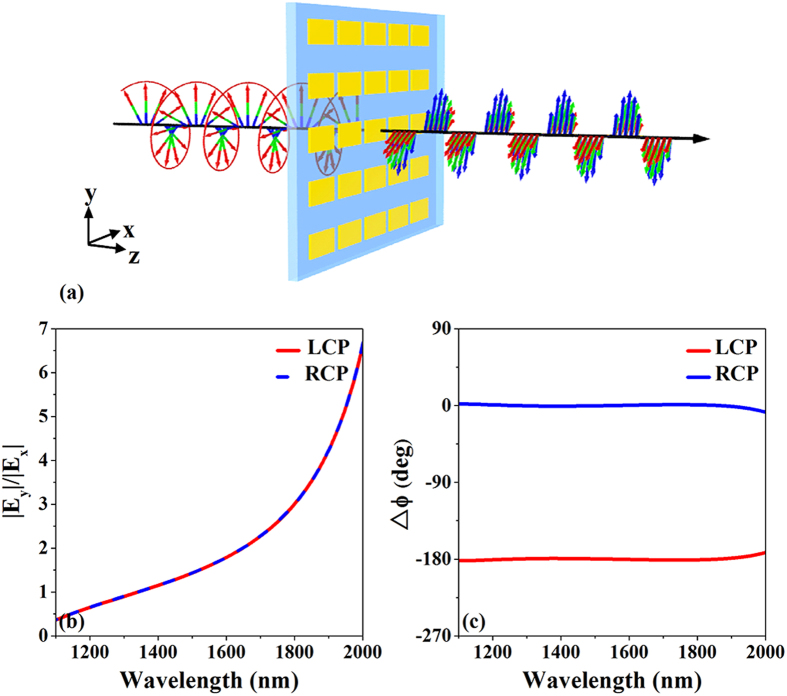
(**a**) Artistic rendering of circular-to-linear (CTL) polarization conversion for the designed ultrathin metasurface. A forward-propagating circularly polarized wave can be transformed into a linearly polarized wave efficiently in broadband. (**b**) Simulated amplitude ratio and (**c**) simulated phase difference of the electric component of the transmitted waves for right- and left-circularly polarized incident waves, respectively, as functions of wavelength.

**Figure 4:**
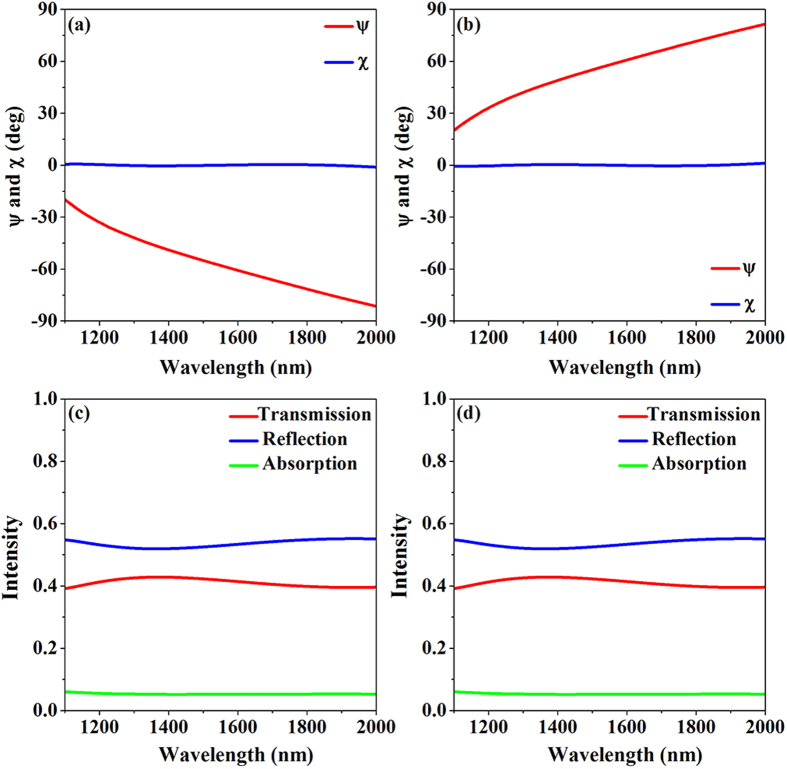
Calculated polarization azimuth angle 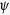 and ellipticity angle 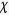 of the transmitted light for (**a**) left- and (**b**) right-circularly polarized incident waves. Transmission, reflection, and absorption spectra for the nanorod metasurface for (**c**) left- and (**d**) right- circularly polarized incident waves.

Figure 3(a) shows an artistic rendering of CTL polarization conversion for the ultrathin metasurface. A forward-propagating circularly polarized wave can be transformed into a linearly polarized wave with a wavelength-dependent electric field polarization angle (the angle between the electric field polarization direction and the *x*-axis) efficiently in broadband. Figure 3(b,c) show the simulated amplitude ratio and phase difference 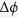 of the electric components of the transmitted wave for right- and left-circularly polarized incident waves, respectively, as a function of the wavelength. Obviously, the ultrathin metasurface satisfies the abovementioned conditions for CTL polarization conversion; the transmitted phase difference 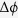 between the electric field components along the *x-* and *y*-axes is exactly −180° or 0° for left- and right-circularly polarized incident waves, respectively. That is, the phase retardation of the electric component of the transmitted wave along the *y*-axis is exactly 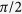. Because the amplitude ratio is wavelength-dependent, the electric field polarization angle of the transmitted wave is dispersive. To investigate the state of polarization of the transmitted wave, the polarization azimuth angle 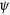 and ellipticity angle 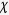 are used to characterize the orientation and shape of the polarization ellipse. Angle 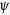 determines the direction of the principal axis of the polarization ellipse, whereas angle 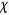 determines the ellipticity. Parameters 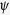 and 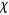 can be obtained from the expressions


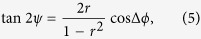



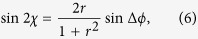


where 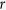 represents the amplitude ratio given by 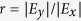. Figure 4(a,b) depict the calculated polarization azimuth angles and ellipticity angles of the transmitted wave as functions of the wavelength for left- and right-circularly polarized incident waves, respectively. The ellipticity angles of the transmitted wave for left- and right-circularly polarized incident waves are exactly equal to zero, meaning that the transmitted wave is ideally linearly polarized, and that this CTL polarization conversion operates in the wavelength range from 1100 nm to 2000 nm. The azimuth angle 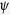 exhibits dispersion, meaning that the principal axis of the polarization ellipse of the transmitted wave changes with the wavelength from −20° to −80° or 20° to 80° for left- and right-circularly incident waves, respectively. The total transmission, reflection, and absorption spectra for left- and right-circular polarized incident waves are shown in Fig. 4(c,d), respectively. The absorption is minimal and the transmission is consistent and over 40% over the whole spectrum; this result proves that the proposed ultrathin metasurface can be used as a lossless CTL polarization converter in the transmission mode.

**Figure 5:**
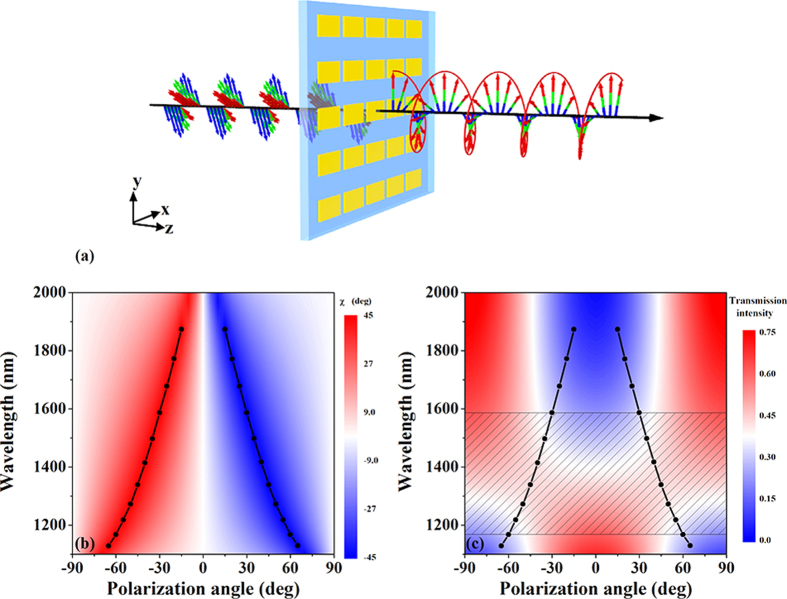
(**a**) Artistic rendering of linear-to-circular (LTC) polarization conversion for the designed ultrathin metasurface. A forward-propagating linearly polarized wave with a wavelength-dependent electric field polarization angle can be transformed into a circularly polarized wave efficiently in broadband. (**b**) Dispersion of ellipticity angle 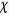 and (**c**) intensity of the transmitted light for a linearly polarized incident wave, showing the influence of the electric field polarization direction of the incident wave on the transmitted wave. The black lines indicate the points with the ellipticity angle 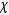 being exactly equal to −45° or 45° with transmission intensities over 30% (as indicated by the regions in gray).

**Figure 6:**
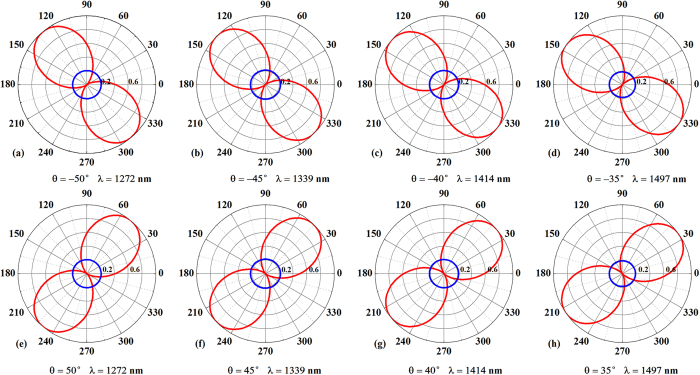
Theoretically predicted polarization states in the plane perpendicular to the wave vector at wavelengths of 1272, 1339, 1414 and 1497 nm for different incident electric field polarization angles (−50°, −45°, −40°, −35°, 50°, 45°, 40°, 35°). The red and blue lines indicate linearly polarized incidence and circularly polarized transmission, respectively.

Due to the dispersion of the amplitude ratio of the electric field of the transmitted wave, the abovementioned conditions of ideal LTC polarization conversion can be satisfied only for a narrow bandwidth for a given electric field polarization angle of the incident wave. An artistic rendering of the LTC polarization conversion for this ultrathin metasurface is shown in Fig. 5(a). By varying the electric field polarization angle of the incident wave with the dispersion of the amplitude ratio, LTC polarization conversion can be realized in broadband. To illustrate the influence of the electric field polarization angle of the incident wave on the transmitted wave, the dispersion of the ellipticity angle 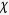 and intensity of the transmitted light in the case of a linearly polarized incident wave are shown in Fig. 5(b,c), respectively. The black lines in Fig. 5(b,c) indicate the points corresponding to the ellipticity angle 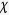 being exactly equal to −45° or 45° with the transmission intensity being over 30% (as indicated by the regions in gray). These results signify that the transmitted wave is exactly circularly polarized and that this ultrathin metasurface can be suitably used as a lossless LTC polarization converter in the wavelength range from 1170 nm to 1590 nm with a wavelength-dependent electric field polarization angle of the incident wave. Furthermore, both left- and right-circularly polarized waves can be obtained for opposite signs of the electric field polarization angle of the linearly polarized incident wave. It is worth mentioning that the conditions for LTC polarization conversion can be satisfied with a 15-nm bandwidth of wavelength in the shadowed regions (in the figure) for a given electric field polarization angle with ellipticity angle 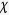 more than −44° or 44° and transmission intensity over 30%, which is also conducive to practical application. Accordingly, the conditions for LTC polarization conversion can still be satisfied for a given incident wavelength when varying the required electric field polarization angle within a range of ±3°. The theoretically predicted polarization states of the incident and transmitted waves in the plane perpendicular to the wave vector at 1272, 1339, 1414, and 1497 nm for different electric field polarization angles of the incident wave (−50°, −45°, −40°, −35°, 50°, 45°, 40°, 35°) are shown in Fig. 6. The red and blue lines indicate linearly polarized incident waves and circularly polarized transmission, respectively. The circular shape of the polarization patterns confirms that the transmitted wave exhibits a high degree of circular polarization, thereby demonstrating that the proposed ultrathin metasurface achieves high-performance lossless broadband LTC polarization conversion.

**Figure 7:**
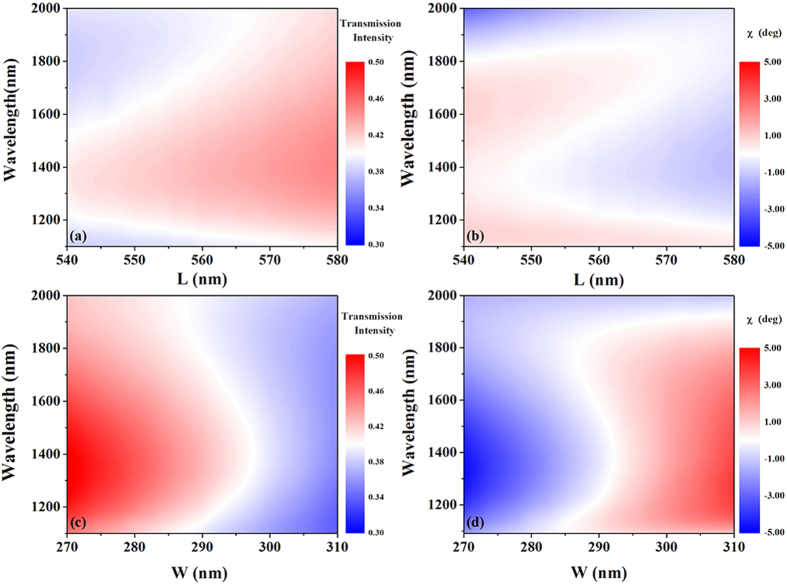
Dispersion of (**a**),(**c**) intensity and (**b**),(**d**) ellipticity angle 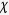 of the transmitted light for right-circularly polarized incidence as a function of the length (L) and width (W) of the nanorod.

Most previously demonstrated polarization converters have been realized via optimization of the structure parameters of a single isolated resonant building block; such a setup is constrained by high-precision requirements of the structure parameters, which is disadvantageous for practical application in terms of the required nanofabrication. The proposed broadband and invertible LTC polarization conversion is realized via induction of the anisotropic optical resonance mode of the gold nanorod, and our simple setup is not constrained by the high-precision requirements of the structure parameters. In this context, in order to examine the influence of the length (*L*) and width (*W*) of the nanorod on the polarization conversion performance, we plot the dispersion of the intensity and ellipticity angle 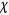 of the transmitted light for a right-circularly polarized incident wave as functions of *L* and *W* in Fig. 7. It is obvious that the dispersion of the intensity and ellipticity angle 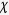 of the transmitted light wave remain steady even when the length or width of the nanorod varies over a fairly large range.

It is worth mentioning that Zhao *et al.* adopted two orthogonally patterned nanorods to achieve polarization conversion from circular to linear^33^, which is mainly realized by the intersection of the two transmission curves between resonant dips. The high-performance lossless linear-to-circular polarization converter in our research is broadband and invertible, which is achieved by the tailoring of the light interference at the subwavelength scale by inducing the anisotropic optical resonance mode of the single nanorod. Moreover, the proposed ultrathin metasurface relaxes the stringent high-precision requirements of the structure parameters in general metasurfaces while retaining the polarization conversion performance.

## Conclusion

In conclusion, we proposed an ultrathin metasurface composed of a single-layer gold nanorod array to achieve broadband and invertible LTC polarization conversion. Our simulated results indicate that the CTL polarization conversion can be realized in the wavelength range of 1100 nm to 2000 nm with a transmission intensity of more than 40%. Further, LTC polarization conversion can be achieved in the range from 1170 nm to 1590 nm with a transmission intensity of more than 30%. Meanwhile, the simulated results show that the LTC polarization conversion conditions can be satisfied with a 15-nm bandwidth of wavelength for a given electric field polarization angle of the incident wave or by varying the required electric field polarization angle within a range of ±3° for a given incident wavelength, which conditions are conducive to practical application. The physical mechanism underlying our broadband and invertible LTC polarization converter is the anisotropic optical resonance mode of the gold nanorod, which ensures that the phase retardation of the electric component of the transmitted wave is exactly equal to 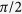 along the y-axis. Our setup for the proposed ultrathin metasurface relaxes the stringent high-precision requirements of the structure parameters in general metasurfaces while retaining the polarization conversion performance. These proposed concepts may find practical applications in novel integrated metasurface-based photonics devices as new platforms for polarization manipulation, optical sensing, and communication functions.

## Additional Information

**How to cite this article**: Li, Z. *et al.* Realizing Broadband and Invertible Linear-to-circular Polarization Converter with Ultrathin Single-layer Metasurface. *Sci. Rep.* **5**, 18106; doi: 10.1038/srep18106 (2015).
